# 3D Printed and Bioprinted Membranes and Scaffolds for the Periodontal Tissue Regeneration: A Narrative Review

**DOI:** 10.3390/membranes12090902

**Published:** 2022-09-19

**Authors:** Irina-Georgeta Sufaru, Georgiana Macovei, Simona Stoleriu, Maria-Alexandra Martu, Ionut Luchian, Diana-Cristala Kappenberg-Nitescu, Sorina Mihaela Solomon

**Affiliations:** 1 Department of Periodontology, Grigore T. Popa University of Medicine and Pharmacy, Universitatii Street 16, 700115 Iasi, Romania; 2 Department of Oral and Dental Diagnostics, Grigore T. Popa University of Medicine and Pharmacy, Universitatii Street 16, 700115 Iasi, Romania; 3 Department of Cariology and Restorative Dental Therapy, Grigore T. Popa University of Medicine and Pharmacy, Universitatii Street 16, 700115 Iasi, Romania

**Keywords:** 3D printing, bioengineering, bioinks, bioprinting, GBR, GTR, scaffolds

## Abstract

Numerous technologies and materials were developed with the aim of repairing and reconstructing the tissue loss in patients with periodontitis. Periodontal guided bone regeneration (GBR) and guided tissue regeneration (GTR) involves the use of a membrane which prevents epithelial cell migration, and helps to maintain the space, creating a protected area in which tissue regeneration is favored. Over the time, manufacturing procedures of such barrier membranes followed important improvements. Three-dimensional (3D) printing technology has led to major innovations in periodontal regeneration methods, using technologies such as inkjet printing, light-assisted 3D printing or micro-extrusion. Besides the 3D printing of monophasic and multi-phasic scaffolds, bioprinting and tissue engineering have emerged as innovative technologies which can change the way we see GTR and GBR.

## 1. Introduction

Periodontitis is a periodontal tissues inflammatory disease, of multifactorial etiology [1,2,3,4], in which the host’s immune response to the aggression of periodontopathogenic bacteria plays a key role [5]. This pathology is characterized by the progressive loss of periodontal attachment, destruction of the alveolar bone, phenomena that, over time, can lead to tooth loss.

Periodontal treatment includes the elimination or modulation of the factors which led to the appearance and evolution of periodontitis, as well as the correction, within the limits of the case and the available technologies, of periodontal soft and hard tissues loss [6,7]. The reconstruction of these defects is essential for the restoration of masticatory, aesthetic, phonation functions and for improving the patients’ quality of life. Therefore, the purpose of complete periodontal therapy is to regenerate the entire periodontal system, which includes bone and cement neo-formation, as well as restoring the attachment of periodontal fibers [8].

The healing following non-surgical periodontal therapy can only provide periodontal repair, represented by the formation of a long epithelial attachment by migrating epithelial cells [9,10]. Therefore, a complete restoration of periodontal anatomy and functionality cannot be ensured [11]. These aspects can be covered by tissue regeneration, with the mobilization and involvement of cellular elements—fibroblasts, osteoblasts and cementoblasts, as well as the signals needed to direct regenerative processes [12].

Guided tissue and bone regeneration (GTR/GBR) are periodontal surgical interventions whose main purpose is to restore the periodontal architecture [13,14]. Primarily, these procedures involve the use of a barrier membrane that may or may not be associated with bone regeneration materials and whose primary function is to prevent epithelial cell migration into the bone defect [15,16]. Moreover, the barrier membrane also serves to maintain the space, creating a protected area in which tissue regeneration is favored. There are four principles considered necessary for successful regeneration, included in the acronym PASS: (P) primary closure of the wound to ensure optimal healing; (A) angiogenesis for adequate vascularization of newly formed tissues, with the supply of oxygen, nutrients, and pro-healing cells; (S) maintaining space for bone neo-formation, preventing inadequate epithelial cell proliferation; (S) wound stability to include blood clot formation [17].

In addition to strength and stability, a number of other characteristics of the barrier membrane can influence the regenerative success. These include pore size, permeability or its architecture [18]. The pore size of barrier membranes has long been a controversial issue. Membrane pore size can influence cell adhesion as well as progenitor cell migration [19]; these processes facilitate not only clot formation but also membrane stabilization, preventing micro-displacements that could disrupt tissue neoformation [20]. Studies have shown that excessively large pores make membranes less effective against soft tissue cells migration and proliferation [21]. To date, an optimal size of barrier membrane porosity has not been confirmed [22].

Over time, there has been a continuous search for ideal materials for tissue regeneration and numerous methods have been developed to stimulate the periodontal tissues repair [23]. The membranes can be classified, depending on their biodegradation capacity, into resorbable membranes (membranes made of synthetic or natural polymers) and non-resorbable membranes (metallic membranes; membranes made of synthetic polymers, polytetrafluoroethylene—PTFE) [24]. Furthermore, another classification includes first generation (non-resorbable), second generation (resorbable) and third generation (membranes as a product of tissue engineering) membranes [25].

GTR and GBR techniques have recently benefited from remarkable advances in the field of three-dimensional (3D) printing, tissue engineering and biofabrication [16,26,27], creating extensive conditions and possibilities for regenerative therapies.

Moreover, 3D printing technology has led to major innovations in periodontal regeneration methods, allowing the printing not only of biocompatible membranes and scaffolds, but also of living cells and supporting components in complex 3D functional tissues, defined as “bioprinting” [28]. The concept of periodontal tissue engineering has thus emerged, being defined as ”the use of physical, chemical, biological, and engineering processes to control and direct the aggregate behavior of cells” [29].

Bioprinting technology is a state-of-the-art tool for rendering biofunctional hierarchical architecture through 3D printing, with one or more types of living cells embedded. Bioprinting refers to the printing of components that form a specific tissue, including living cells embedded in matrix materials, to generate analogous tissue structures [29]. Bioprinting techniques bring a biological functionality to a conventional 3D printed scaffold, as it mimics a cell-to-cell and cell-to-matrix interaction in construction [30]. The material used in 3D bioprinting involves the usage of living biological cells, hydrogels, chemical factors and biomolecules, under the name of ”bioink”. A difference must be made regarding the types of printing material, between ”bioinks” and ”biomaterial inks”, as those two terms do not assimilate each other. While a bioink is cell-laden, acting as a cell carrier and deliverer during formulation and bioprinting processing, biomaterial inks are cell-free and can only be seeded with cells after printing [31].

The aim of this paper is to review the present data in the literature, related to 3D printing and bioprinting techniques and materials, as well as applications in the form of membranes and scaffolds in the regeneration of the periodontal apparatus.

## 2. 3D Printing and Bioprinting Techniques

**Figure 1:**
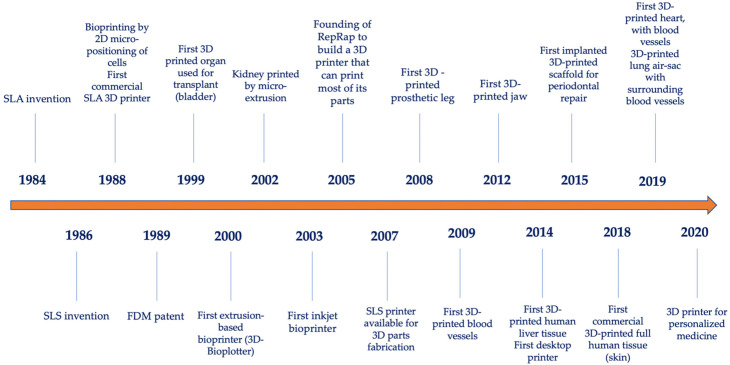
The evolution of 3D printing technologies.

3D printing is a revolutionary technology also known as additive manufacturing [29]. Figure 1 shows a series of milestones in the chronological evolution of 3D printing and bioprinting technologies. In 1984, Charles Hull invented stereolithography (SLA) for printing 3D objects from digital data [32]. In 1986 Carl Deckard and Joe Beaman invented selective laser sintering (SLS) [33].

Bioprinting was first demonstrated in 1988 by Klebe with a standard Hewlett-Packard (HP) inkjet printer to deposit cells by cytowriting technology [34]. Moreover, in 1988, the first SLA 3D printer was available [35]. In 1989, the fused deposition modeling (FDM) technique was developed by Scott Crump [36]. In 1999, the first organ used for transplantation (bladder) was printed [37].

In the year 2000, the first extrusion-based 3D printer (3D-Bioplotter) appeared, and two years after that, the first kidney was printed by micro-extrusion [38]. In 2003 Wilson and Boland developed the first inkjet bioprinter by modifying a standard HP inkjet printer [39]. In 2005, the RepRap (Replicating Rapid prototyper) project was founded at the University of Bath, Somerset, UK, with the aim of creating a low-cost 3D printer that would print most of its components [40]. The first SLS-based 3D printer was available in 2007 [41].

The first prosthetic limb was printed in 2008 [42] and the first blood vessels were 3D printed in 2009 [43]. The first 3D printed jaw was made in 2012 [44]. The year 2014 marks the first 3D printing of liver tissue [45] but also the appearance of the first desktop 3D printer [46]. Rasperini et al. developed and applied the first 3D-printed scaffold for periodontal repair in 2015 [26]. In 2018, the first commercial 3D-printed full human (skin) product became available [47] and in 2019, Noor et al. succeeded in generating a perfusable heart with reduced dimensions [48]. The 2020s pave the way for the personalized use of 3D printing in medicine [49].

3D printing technology has found numerous applications in dentistry and periodontics, in particular. 3D printing methods have been investigated in the treatment of gingival lesions (treatment of gingival recessions, gingivectomies or restoration of the smile design), as well as regeneration of periodontal tissues: alveolar bone, periodontal ligaments (PDL) and cement [50,51]. Most commonly used biomaterials in periodontal tissue regeneration 3D assisted are presented in Table 1, along with their main advantages and disadvantages.

The main 3D printing techniques in GTR and GBR include droplet-based printing, micro-extrusion and light-assisted printing [28].

### 2.1. Droplet-Based Printing

Droplet-based 3D printed products are the result of independent and discrete droplets as basic unit. These techniques can be divided inro inkjet 3D printing and electrohydrodynamic jetting. Moreover, inkjet printing can be performed either by continuous inkjet printing or drop-on-demand printing [43].

Inkjet printers used to be the most frequently approached method in both non-biological and biological applications [28]. The principle of operation involves the controlled passage of a low-viscosity solution flow, an absolutely necessary condition due to the excessive force required to release droplets when using solutions at higher viscosities [52]. The fluid subsequently breaks into very small-volume droplets (1–100 pL) after passing through the holes in the printhead; these droplets are then distributed under the influence of pressure pulses in predefined locations, to form three-dimensional structures after solidification [53].

The development of inkjet printers in biological printing is based on classic, two-dimensional inkjet printers [28]; in the modified version, the ink, the cartridge and the paper are replaced with biocompatible materials and the control is performed three-dimensionally, in the three axes (OX, OY and OZ) [54]. Subsequently, printheads with several hundred individual nozzles were developed [11].

**Figure 2:**
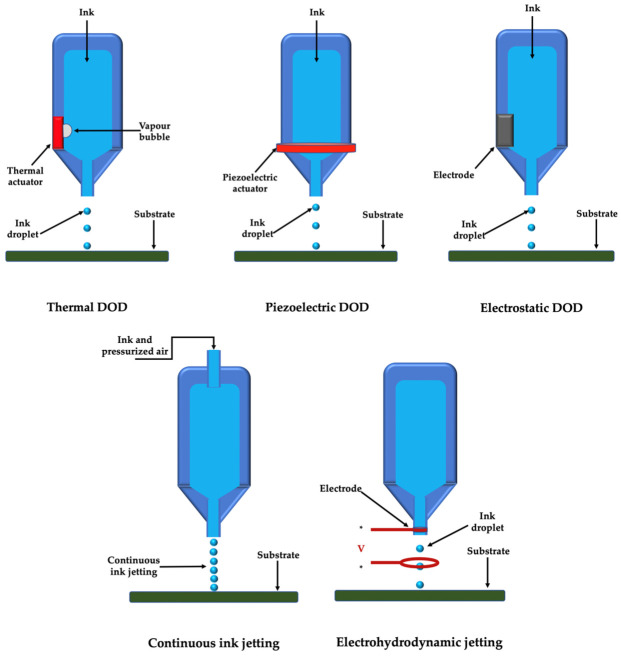
Droplet-based 3D printing techniques; DOD: drop-on-demand.

Continuous inkjet printing is based on a natural phenomenon called Rayleigh-plateau instability, which exhibits the natural tendency for a stream of liquid to undergo a morphological transformation to a train of discrete drops (Figure 2). On the other hand, drop-on-demand printer produces a droplet when required; droplet deposition is performed by displacing the nozzle above the desired location before a droplet is ejected [43]. Modulation and conditions of drop-on-demand printing can also be done thermally, acoustically or electromagnetically (Figure 2).

A number of major disadvantages, however, make the use of inkjet printers limited. In the case of thermal inkjet printers there is an increased risk of thermal and mechanical stress; moreover, low directionality and uneven droplet size, as well as frequent nozzle clogging, have been reported [55]. The disadvantages encountered in the case of thermal inkjet printers have tried to be overcome by introducing acoustic inkjet printers or electromagnetic inkjet printers [56]. The concern in the case of electromagnetic inkjet printers is related to the used frequencies (15–25 kHz) which can induce damage to the cell membrane [57].

Moreover, a high pressure is required when using inkjet printers, an aspect which can affect the cell viability within the bioink. In order to counteract this particular disadvantage, electrohydrodynamic jetting avoids such pressures by using an electric field (Figure 2) [58]. Furthermore, electrohydrodynamic jetting is particularly suitable for printing bioink with high cell concentration and weight/volume ratio.

The metallic nozzle is filled with bioink and a spherical meniscus is formed at the tip of the nozzle due to surface tension. A high voltage is applied between nozzle and substrate and droplets are ejected when the electrostatic stresses overcome the surface tension under a sufficiently high voltage [59]. Different voltages will generate different types of inkjets (micro-dripping, intermittent jetting, breakdown, etc.), but the most frequently used are independent, discrete droplets [43]. Also, higher voltages will produce smaller droplets [60].

### 2.2. Light-Assisted 3D Printing

Light-assisted 3D printing uses laser-assisted, photocuring-based or stereolithography techniques in order to generate printed and bioprinted products.

Laser-assisted printing involves nozzle-free and non-contacting techniques, which include laser-induced forward transfer (LIFT), laser-induced forward transfer supported by an absorption film (AFA-LIFT), matrix-assisted laser evaporation direct writing (MAPLE-DW), biological processing (BioLP) or laser guidance direct writing (LGDW).

**Figure 3:**
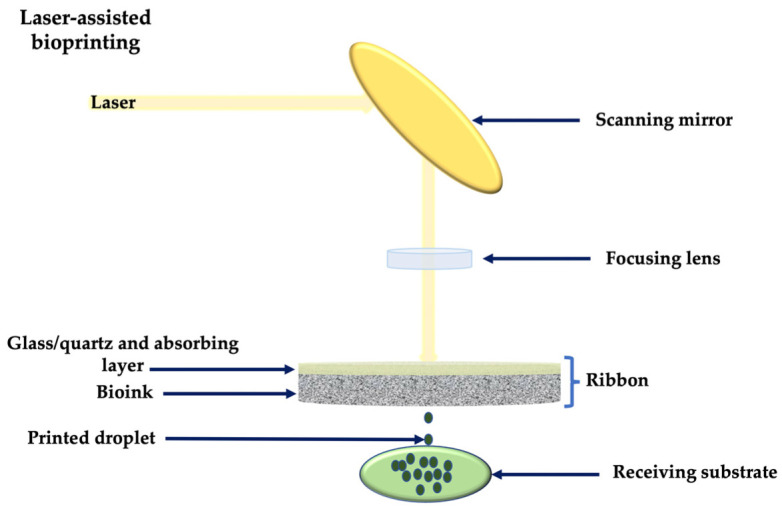
Laser-assisted bioprinting.

Generally, a laser-assisted printer is composed of: a pulsed laser source, usually a nanosecond laser with ultraviolet wavelength; optics necessary for the beam delivery; a target in the form of a glass/quartz ribbon coated with the bioink; and a receiving substrate coated with biopolymer or cell medium (Figure 3) [31]. The laser beam is directed at the absorbing layer of the ribbon, generating local evaporation and the formation of high-pressure bubbles, propelling the cell-containing material towards the receiving substrate [43]. Laser parameters need to be accurately set, in order to control the usual photothermal, photochemical and photomechanical laser effects.

Laser-assisted techniques allow bioprinting with biomaterials of high cell density and viscosity, at a high resolution. Being nozzle-free instruments, they also avoid clogging or high shear stress problems generated by other techniques [61]. Their main disadvantages include the high cost and the complex printing technique.

Stereolithography (SLA) was the first commercially available 3D printing technology. The basic principle of SLA is given by the photopolymerization of a highly crosslinked viscous polymer or prepolymer under exposure to a light energy source (laser or UV), to create layered structures [62].

**Figure 4:**
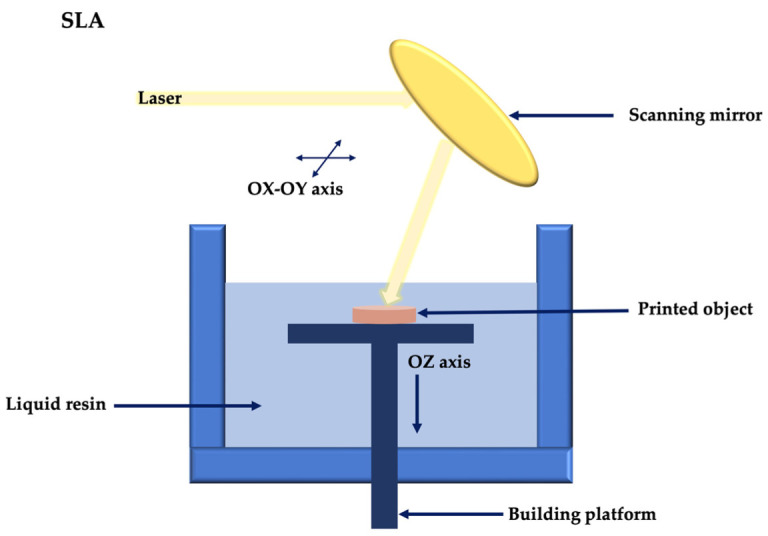
Stereolithography schematic principle.

The SLA printing process involves three main stages: exposure to the light radiation source, platform movement, and polymer filling (Figure 4) [63]. The polymerization layers are bonded from the bottom up, following the exposure of the resin to light radiation; as one layer is polymerized, the platform descends for a distance equal to the thickness of one layer and builds the next layer until the printing of the digitized 3D object is completed [63,64]. In order to be used in regenerative therapy, SLA involves the use of a slowly photopolymerized prepolymer formulation, loaded with cells [64].

**Figure 5:**
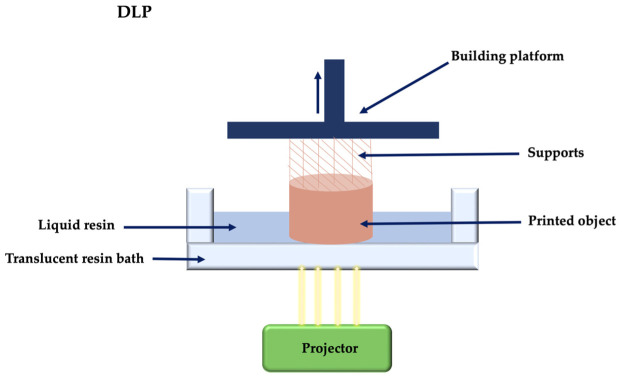
Digital projection printing principle.

Digital projection printing (DLP) is similar to SLA procedure, with the difference that, while in the case of SLA the light beam moves, in the case of DLP it is stationary, which makes DLP a process with a higher printing speed, but less accurate [63]. DLP uses liquid photosensitive resins which, under the action of light curing, will form the 3D construction, layer by layer (Figure 5). DLP printers use a system of micro-mirrors that conduct light to the projection lens [65].

**Figure 6:**
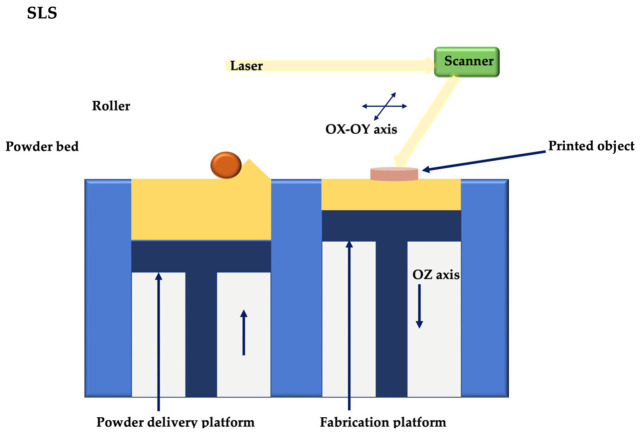
Selective laser sintering principle.

Selective laser sintering (SLS) uses a high energy laser beam to induce the fusion of the raw material in powder form, layer by layer. SLS does not require additional material support during printing because the support is provided by the surrounding powder (Figure 6) [66]. This particular technique uses a high energy laser which selectively fuses the powdered material by scanning cross-sections generated from the digital file.

### 2.3. Extrusion 3D Printing

Of the various 3D bioprinting technologies, extrusion is the most commonly used in medical applications [57]. Extrusion 3D printing can be performed under thermal and non-thermal conditions.

#### 2.3.1. Thermal Extrusion 3D Printing

**Figure 7:**
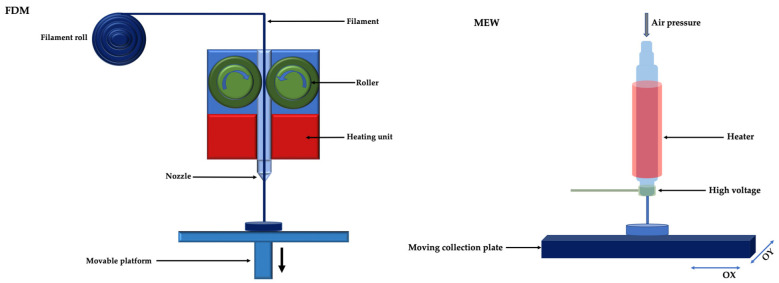
Thermal extrusion 3D printing: fused deposition modelling (**right**) and melt electrowritting (**left**).

Thermal extrusion 3D printing can be done by fused deposition modelling (FDM) or melt electrowriting (MEW) (Figure 7). Fused deposition modelling (FDM) is based on layer-by-layer printing of thermoplastic polymers. The polymer is heated over its melting point and, due to a solid-semisolid transition, is then extruded into filaments with diameters of 160–700 μm, through a nozzle, according to the predefined design [16]. FDM is characterized by high printing speed; in addition, by adding multiple nozzle heads, various materials can be printed simultaneously [67]. Due to the fact that FDM uses high temperatures (140–250 °C), it cannot be used in bioprinting; this technique can be approached, however, for the realization of multiphase scaffolds of periodontal regeneration [68,69,70].

The main disadvantages of FDM include the low resolution and the surface finish that may be poor, following the spread of the material before it has cooled; it should be noted, however, that these drawbacks can be overcome by decreasing the nozzle diameter [71].

Melt electrowriting combines FDM with the electrospinning technique to obtain extremely thin filaments (generally 2–30 μm), even nanometric [72], directed in porous constructions with high degree of complexity and precision. MEW involves applying an electric field to continuously pull a molten polymer toward a computer-controlled static or rotating collector plate [73]. The essential parameters in the realization of such constructions are represented by the delivery rate of the polymer, the needle diameter, the collector speed, the distance from the needle tip to the collector, as well as the applied electrical voltage [16,74]; by adjusting these variables, fibers can be obtained on a scale very close to native collagen fibers [75]. Thus, MEW is characterized by a remarkable resolution of the fibers, with the ability to print 1 cm thick structures, which makes this technique ideal for scaffolding or cell bioprinting. To date, most research has focused on prints in the same plane as the substrate/construction plate; it has been shown, however, that constructions in anatomical forms can be obtained from MEW using biomaterials such as hydrogel or bioceramics [76].

#### 2.3.2. Non-Thermal Extrusion 3D Printing

**Figure 8:**
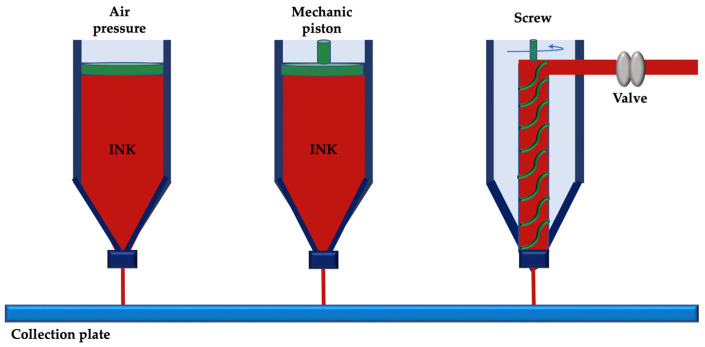
Non-thermal extrusion 3D printing.

Non-thermal extrusion techniques allow 3D printing by extrusion without melting the material. This printing system can use air pressure, a pressurized piston without a valve or a screw to extrude cell-free or cell-loaded biomaterials on a substrate, avoiding the distortion of temperature-sensitive biomolecules and/or cell death (Figure 8) [16].

Extremely important parameters in this technique are represented by the properties of the material, the quantity and quality of the used additives. Non-thermal extrusion has been used mainly for making cell-loaded scaffolds at physiological temperatures [77].

Although significant progress has been reported in extrusion-based 3D bioprinting, further improvements in bioink availability are needed; hydrogels with suitable printing properties have been developed, which can ensure a maintenance of the post-printing 3D shape without compromising the viability of the obtained product [78].

## 3. Applications of 3D Printing in Periodontology

### 3.1. 3D Printed Scaffolds in Periodontal Defects

**Figure 9:**
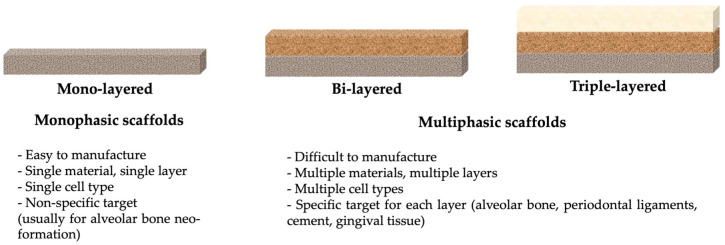
Schematic view of monophasic and multiphasic scaffolds.

Monophasic scaffolds are characterized by the presence of a single compartment, having the characteristic functions of a barrier membrane: maintaining and stabilizing the bone defect subject to regeneration; ensuring bone proliferation, without epithelial interference in bone defect; control of the healing process in time and space [79]. Moreover, these scaffolds can be loaded with growth factors or cells to promote bone neo-formation [11]. Obtaining alveolar bone tissue, facilitated by monophasic scaffolds, is not sufficient for restitutio ad integrum. Thus, biphasic scaffolds have been developed for the periodontal ligaments’ regeneration. Triphasic scaffolds have emerged in order to promote the regeneration of cementum, together with the alveolar bone and periodontal ligaments. A schematic structure of printed scaffolds, along with their main characteristics, are presented in Figure 9.

Carrel et al. [80] developed an extrusion scaffold, consisting of cylindrical filaments of tricalcium beta-phosphate (β-TCP) and hydroxyapatite (HA), arranged in orthogonal layers (OsteoFlux^®^). It was placed in sheep calvary defects and was compared with bovine bone (Bio-Oss^®^) and β-TCP particles. In the first 8 weeks the authors observed a significant increase in bone growth, but no difference was registered in the total of four months [80].

Another scaffold, consisting of 30% HA, 60% β-TCP and 10% tricalcium alpha-phosphate (α-TCP), also made by extrusion, in the form of a mesh, with a macroporosity of 60%, was implanted in sheep sinus [81]. The scaffold was well tolerated, generating a peripheral bone remodeling in the first 45 days after implantation; at 90 days, the formation of a peripheral lamellar bone was observed, but after 90 days the scaffold continued to resorb, leaving an incompletely filled bone defect, with areas of fibrous tissue [81].

Cellular loading of hydrogel scaffolds was also attempted, mainly by electrospinning techniques; these forms of scaffolds have, however, important limitations related to cell source and culture [11,82,83]. Improved single-phase scaffolds with growth factors have also been developed. Cho et al. [84] used extrusion to make a polycaprolactone (PCL) scaffold, loaded with poly (lactic-co-glycolic acid) (PLGA) microspheres with morphogenetic protein 2 and 7 (BMP-2, BMP-7) and connective tissue growth factor (CTGF); the scaffold was implanted in vitro on the root surface of human teeth with the removed cementum, in a cementogenic/osteogenic environment. After 6 weeks, all groups delivered with growth factor showed surface recovery of the dentin with a layer similar to the newly formed cement, compared to the control. BMP-2 and BMP-7 showed de novo formation of significantly thicker tissue layers than all other groups, while CTGF and BMP-7 resulted in significantly improved integration on the dentin surface [84].

Shim et al. [85] compared 3D printed polycaprolactone (PCL) and tricalcium polycaprolactone/β-phosphate (PCL/β-TCP) membranes with a conventional commercial collagen membrane in terms of their ability to facilitate GBR, investigating the mechanical properties in dry and humid environment. Fibroblasts and pre-osteoblasts were seeded in membranes and proliferation rates and patterns were analyzed with scanning electron microscopy. Subsequently, the membranes were placed in alveolar defects in beagle dogs. CT and histological analyzes at eight weeks postoperatively showed that 3D-printed PCL/β-TCP membranes were more efficient than 3D-printed PCL and substantially better than conventional collagen membranes in terms of biocompatibility and bone regeneration [85].

Dubey et al. [86] designed, by infusing a PCL mesh made by MEW, with a hydrogel loaded with amorphous magnesium phosphate (AMP), a membrane reinforced with highly adjustable bioactive fibers for GBR. This membrane proved that the presence of PCL networks manufactured by MEW can delay the degradation of the hydrogel; thus, soft tissue invasion is prevented. At the same time, a mechanical barrier is generated to allow slower-migrating progenitor cells to participate in bone regeneration [86].

Hsieh et al. [87] investigated the biological characteristics of human PDL cell spheroids formed on chitosan and polyvinyl alcohol; PDL cell spheroids were cultured in 3D-printed polylactic acid scaffolds by FDM to assess mineralization capacity. Cellular spheroids formed on the chitosan membrane demonstrated an increased alkaline phosphatase activity, as well as an increase in mineralized matrix deposits [87].

Bai et al. [88] have developed an individualized titanium mesh (Ti-Mesh) using computer-aided design and sintering additive manufacturing technology to evaluate the effect of different thicknesses and sizes of titanium mesh pores on its mechanical properties. The authors observed that, as the mesh diameter increased (3 mm to 5 mm), the mechanical properties of the mesh decreased. The 0.4 mm thick titanium mesh proved to be strong enough with little mucosal stimulation.

Porous networks of 3D printed titanium-niobium alloy (Ti-Nb) have also been developed in order to maintain the space, to prevent the fibroblasts’ growth and inhibit the bacterial colonization [89]. In this technique, Ti-Nb alloy meshes were prepared by selective laser melting (SLM) and used as substrates for new surface coatings. Porous coatings of chitosan (CS)/gelatin (G)/doxycycline (Dox) were formed on the meshes, using electrophoretic deposition (EPD) and lyophilization. This membrane has been shown to be effective in preventing the growth of fibroblasts, while allowing nutrient infiltration; moreover, its antibacterial effect was observed.

A scaffold with two compartments was designed: bone and ligament, by combining extrusion with casting; the wax forms were made by extrusion, and the materials were then cast into these shapes [90]. The bone compartment was seeded with Ad-CMV-BMP7 transduced periodontal ligament cells and the ligament compartment, composed of three superimposed cylinders, was seeded with PDL cells. The authors observed that parallel and oblique oriented fibers were generated, which grew and traversed the designed PCL and poly (glycolic acid) (PGA) structures, forming ligaments and bone structures similar to the native ones; the disadvantage, however, is the inability to control and predict cell directionality in vivo [90].

Vaquette et al. designed a scaffold with a bone compartment made by β-TCP FDM, seeded with osteoblasts, and a ligament compartment produced by electrospinning, in which PDL cell sheets were placed [69]. Following the in vitro test, it was observed that osteoblasts formed a mineralized matrix in the bone compartment after 21 days of culture and that harvesting the PDL cell sheet did not induce significant cell death. The biphasic scaffold seeded with cells was placed on a block of dentin and implanted for 8 weeks in a subcutaneous model of athymic rat. The authors observed bone neoformation, ligament and cement regeneration (but with perpendicular non-orientated non-functional periodontal fibers) [69]. This model was taken over and modified by Costa et al. [91]; the bone compartment, with an increased pore size, was covered with a layer of calcium phosphate (CaP) to increase osteoconductivity, seeded with osteoblasts and cultured in vitro for 6 weeks. Then PDL cell sheets were placed in the obtained product, subsequently implanted subcutaneously in athymic rats for 8 weeks. These modifications led to better bone neo-formation, better oblique orientation of periodontal fibers (but poorly controlled) and increased vascularization [91].

Wang et al. [92] developed a biphasic scaffold model made of collagen and strontium-doped calcium silicate, manufactured by extrusion, loaded with gingival fibroblast cells; it was placed in calvary defects in rabbits. The authors found that biphasic scaffolds improved osteogenesis [92].

Lee et al. developed a triphasic scaffold with a precise architecture, enriched with biochemical gradients [70]. This scaffold was made up of three distinct, layered compartments, corresponding to the morphology of the periodontal complex: cementum, periodontal ligament and alveolar bone. Each component had a specific architecture, with variable pore sizes (100, 600 and 300 μm). Moreover, PGA microspheres loaded with growth factors specific to the regeneration of each tissue type (amelogenin, CTGF and BMP-2) were added to each compartment. The scaffold was digitally designed and 3D printed, but the addition of microspheres was done manually, by pipetting. The authors observed a discontinuous cementogenesis, with a notable osteogenesis in the bone compartment. Interposed connective tissue between these two mineralized formations was found, with an alignment of the fibers and a ligament attachment on the newly formed cementoid tissue [70].

Rasperini et al. [26] designed a customized scaffold, based on the patient’s computed tomography; the scaffold was made of PCL powder containing HA, using selective laser sintering. Although this scaffold proved effective in treating large periodontal defects, at 12 months it became exposed, which compromised the therapeutic result.

Therefore, triphasic scaffolds, with a three-compartment design, have an important potential in complete periodontal regeneration, which involves the formation of cement on the root surface, the formation and proper insertion of periodontal fibers in hard tissues and sufficient rigidity in general [93], but their design and implementation require further investigation.

One concern in making and functionalizing multiphase scaffolds was poor interphase cohesion, which affects the mechanical stability of the scaffold. Continuous additive fabrication or simultaneous multiphase crosslinking seem viable options to overcome this dilemma [94,95].

### 3.2. Socket Preservation

Bone resorption after dental extraction represents a common challenge and various techniques have been developed and investigated, 3D printing included, in order to preserve an adequate height of the alveolar bone. Park et al. [96] evaluated the efficiency of a 3D printed PCL scaffold implanted in artificially created saddle-type bone defects in beagle dogs, along with β-TCP. The authors observed that the PCL scaffold maintained the physical space, without any inflammatory infiltrates around the scaffold.

Goh et al. [97] inserted a 3D bioresorbable PCL scaffold in fresh extraction sockets in a randomized controlled clinical trial (RCT). The micro-CT and histological analysis revealed that this treatment option resulted in a lower vertical ridge resorption at the 6-months evaluation.

3D printing was used also for HA granules manufacturing, which were placed in alveolar sockets after atraumatic extractions and covered with collagen membrane [98]. At the 8-weeks evaluation, the grafted site was completely filled with woven bone, with vascular neo-formation and without any inflammatory signs.

### 3.3. Other Applications

Positive outcomes have been observed in bone augmentation for sinus lift procedures when using 3D applications [99]. Different materials have been tested, such as monolithic monetite (dicalcium phosphate anhydrous) or biphasic calcium phosphate [100]. The main advantages of using 3D printed products for sinus lift are represented by the low risk of infection transfer, as well as by shorter surgery time and higher comfort for the patient [101]. A synthesis of the main 3D applications in available studies is presented in Table 2.

Other applications include 3D-surgical guide precise implant placement, with both static and dynamic implantation guiding [111,112,113,114,115,116,117,118]. 3D printing has been also extensively used in education of dental school students and residents and even for patient motivation.

## 4. Bioprinting

The 3D bioprinting process comprises six major stages:

- Data acquisition, using X-ray scanning and reconstruction techniques, computed tomography (CT), magnetic resonance imaging (MRI), or directly using computer-aided design (CAD) software. These data will be processed with the help of specific software. The file is converted to a printer-readable file [119]. The data is then translated to allow estimation of the amount of material to be extruded, which depends on the desired height and width of the layer according to the shape of the bioink (droplet or filament) [31,120].
- The choice of bioink, which is made according to the printing technique and the requirements of the printed structures. Thus, the bioink must meet favorable mechanical properties, as well as biocompatibility and printability requirements. The bioink can contain isolated cells, growth factors and bioprinting materials. It is prepared according to the physiological temperature, pH and requirements of the printed structures [31].
- Setting the appropriate printing parameters, depending on the bioink and the desired structure of the printed product.
- The actual bioprinting, under close observation to make adjustments when necessary. Printing resolution is specific to the printer and the type of bioink. In cases of high resolution, the time to fabricate the object can be longer [121].
- Post-printing stage, which can include spinning and microscopical assessment of the printed object. The bioprinted object is kept in an incubator or bioreactor.
- Placement of the bioprinted product (in vivo or in vitro conditions).

3D bioprinting involves precise, layer-by-layer positioning of biological materials and living cells [57]. Some applications of 3D bioprinting include stem cell research [122], anti-cancer therapy [123], drug testing [124] and tissue engineering [125]. In tissue engineering, these technologies can control pore size, shape, distribution and interconnectivity. Moreover, by associating bioprinting technologies with imaging tests, such as cone-beam computed tomography (CBCT), specialized constructions can be made, adapted to the case and site-specific [126].

Bioprinting is based on three fundamental concepts: biomimicry, self-assembly and building blocks with mini-tissue [127]. Biomimicry involves the creation of exact replicas of the cellular and extracellular parts of a tissue and organ [128]. So far, bioprinting has been adopted for the manufacturing of bioartificial tissues and organs, such as skin, bones, cartilage, kidneys, heart, lungs [53,103,129,130,131,132,133]. Self-assembly involves the design of a scaffold-free method that mimics the behavior of embryonic stem cells and mini-tissues can be defined as the smallest structural and functional component of a tissue [130]. 

Although this concept is still in its infancy, a number of in vitro and in vivo studies have shown interesting perspectives that go beyond the principles of barrier membranes and scaffolds. At the same time, there are numerous limitations to overcome, which include production technology and bioinks optimization [134]. Photo-crosslinkable hydrogels, gelatin-methacryloyl hydrogel and poly (ethylene glycol) dimethacrylate have been proposed as basic materials in bioprinting, and the optimization of printing capacity, mechanical stability and cytocompatibility has been tried by testing different extrusion parameters and crosslinking methods [134].

Wang et al. [135] proposed an active tissue engineering scaffold for the repair of bone defects. This material was constructed with a 3D-BG scaffold composite, MSC and nanoparticles loaded with the BMP-2 gene (BMP/CS). The scaffold was subsequently implanted in the alveolar bone defect of rhesus monkeys and its capacity for osteogenesis was assessed. The authors found that this scaffold model promoted bone healing, with enhanced osteogenic properties in vivo [135].

Lin et al. [136] developed a biomimetic microfiber system, able to withstand functional load, to help regenerate PDL. Straight, collagen-based waveform microfibers to guide the growth of PDL cells were prepared by extrusion and a laminar flow-based bioreactor was used to generate fluid shear stress. PDL cells were seeded on those microfibers [136]. The authors found that microfibers maintained the viability of PDL cells and showed an improved tendency to promote healing and regeneration under shear stress.

Vurat et al. [137] developed a multicellular micro-tissue model similar to the periodontal ligament-alveolar bone biointerface. The periodontal ligament layer was modeled using methacryloyl gelatin bioink (Gel-MA) and ligament fibroblasts. The alveolar bone layer was modeled using a composite bioink composed of Gel-MA and HA-magnetic iron oxide nanoparticles (Gel-MA/HAp-MNPs), and osteoblasts [137]. The bioprinted self-supporting microfabric was cultured under flow in a microfluidic platform for more than 10 days, without significant loss of shape fidelity. Confocal microscopy analysis indicated that the encapsulated cells were homogeneously distributed inside the matrix and maintained their viability for more than 7 days under microfluidic conditions. Preliminary interaction study with tetracycline indicated absorption of the drug by cells inside the 3D microfabric [137].

Li et al. [138] developed a system for vascularized bone regeneration; they combined ethosomes loaded with deferoxamine (DFO) (Eth) with Gel-MA/gellan gum methacrylate (GGMA) to fabricate 3D printed scaffolds by photo and ionic crosslinking. This system had good cytocompatibility, generated sustained release of DFO, which significantly promoted endothelial cell migration and tube formation, mineralized matrix deposition, and alkaline phosphatase expression [138]. The scaffold was later placed in a rat cranial defect; the signaling pathway of inducible hypoxia factor 1-α (HIF1-α) has been activated, indicating that the composite scaffold could promote angiogenesis and bone regeneration [138].

Although 3D bioprinting has been identified as a technology capable of changing the paradigms of regenerative and reconstructive periodontal therapy, it still finds itself at the beginning of the road. Of course, further studies to translate in vitro models to experimental animals and, subsequently, why not, to human subjects, are an absolute necessity for the establishment of manufacturing and implementation protocols.

## 5. Future Directions

Periodontal regeneration involves a high degree of intricacy due to the complex nature and architecture of the periodontal apparatus. The coordination of the components, gingiva, periodontal ligaments, alveolar bone and root cementum, is of crucial importance in the complete regeneration of periodontal structures. In addition, the periodontal system is susceptible to microbial flora, as well as to local and systemic risk factors for periodontal disease. Translating in vitro and in vivo models into clinical models on human subjects is the main challenge associated with the development of technologies. Moreover, the present studies pay little attention to antimicrobial and immunomodulatory strategies. The integration of antimicrobial drugs and/or biomaterials with immunomodulatory capacity is an extremely interesting future direction.

An interesting approach that is still in its early stages is the reconstruction of highly organized living tissues and tissue interfaces in complementary converged 3D printing/bio-printing technologies, integrated into a single biofabrication platform, called multi-technology manufacturing. This field can generate scaffold systems with high print fidelity, spatial control over cell distribution and improved biomechanical properties, without compromising cell viability and functionality [139].

Studies already present in the literature have investigated the association of hydrogel printing with MEW [140]. The authors combined extrusion-based bioengineering and MEW in a single biofabrication platform, which allowed the fabrication of living constructs with spatial distribution of mesenchymal stromal cells, with improved functionality and biomechanics, without compromising cell viability or chondrogenic differentiation [140]. Diloksumpan et al. [141] also used convergent technologies to achieve hard-soft tissue interfaces, using hydrogel and bioceramics.

Dos Santos et al. [142] combined co-axial electrospinning and 3D printing techniques to generate zein-based bilayers as a potential platform for dual delivery of periodontal tissue regeneration drugs. In vitro experiments showed that the two-layer constructs provided sustained release of distinct drugs for 8 days and showed biocompatibility against human oral keratinocytes (Nok-si) (cell viability > 80%), as well as antibacterial activity against bacterial strains. distinct, including those from the red complex, such as *Porphyromonas gingivalis* and *Treponema denticola* [142].

Liu et al. [143] associated directly deposition 3D printing of PCL/gel/nano-hydroxyapatite (n-HA) scaffolds with polycaprolactone/gelatin nanofiber membranes (PCL/Gel) by electrospinning. The authors noted that after 20 weeks, the PCL/Gel/n-HA scaffold-treated sites showed a higher degree of new bone formation than in the control group, indicating that this is a favorable combination of GBR materials [143].

The fourth dimension was introduced in the bioprinting field; thus, smart scaffolds have emerged, programmed to undergo shape or functional changes according to the desired stimulation in time [144]. 3D-printed networks are stimulated by the environmental factor to fold into tubes, mimicking vascular-like tissue constructs [31].

Other promising technologies include external magnetic field stimulation [145] or atmospheric plasma functionalization [146]. Also, new perspectives are brought by real-time monitoring of the printing process through combinations of artificial vision, inspection sensors and feedback control systems supervised by high-efficiency artificial intelligence algorithms and the biofabrication of truly functional tissue equivalents [16,139].

## 6. Conclusions

In response to the requirements of periodontal GTR and GBR, new feeding materials and 3D printing methods have been developed, with the aim of accelerating production and improving performance, with favorable in vitro and even in animal models results. These aspects propel membrane printing and multiphasic scaffolds as a new and promising approach in the reconstruction of periodontal tissues.

Of course, many limitations related to the bioavailability of bioinks, the mechanical properties of the printed structure and its dimensional accuracy are still to be overcome. Moreover, translating the obtained models to human subjects remains an important challenge. Improving digitally assisted techniques and biomaterials, together with the combination of CBCT investigations, can facilitate this translation, with the production of patient- and site-specific scaffolds.

Tissue engineering is still in its early-stage development, due to the lack of in vivo and clinical evaluation in periodontal defect models, the available data being limited.

## Figures and Tables

**Table 1:** Main biomaterials used in scaffold 3D printing.

Material	Advantages	Disadvantages
**Natural polymers** Collagen Alginate Hyaluronic acid Chitosan	Biocompatible Good cell affinity Hydrophilicity Antibacterial effect	Low mechanical properties Fast degradation rate Lack of bioactivity
**Synthetic polymers** Polycaprolactone (PCL) Polylactic acid (PLA) Polyglycolic acid (PGA) Polyethylene glycol (PEG) Poly(lactic-co-glycolic) acid (PLGA)	Highly adjustable physiochemical and mechanical properties Wide range of degradation and resorption kinetics Good repeatability	Low bioactivity Slow degradation rate Acidic byproducts
**Bio-ceramics** Hydroxyapatite (HA) β-tricalcium phosphate (β-TCP) Bioactive glass	Bioactive Biocompatible Osteoconductive Potential osteoinductive Hydrophilicity	Not compatible with cell encapsulation Stiffness Brittleness Low ductility Low flexibility Inconsistent cell reactions (variations in surface quality)

**Table 2:** Applications of 3D printing in periodontology.

Application	Authors	Type of Study	Method	Material	3D Printer
GTR	Kim et al., 2010 [52]	In vivo	3D-printed tooth scaffold	Poly-epsilon caprolactone and hydroxyapatite	Not mentioned
	Park et al., 2010 [90]	In vivo	3D-printed scaffold	PCL-PGA	3D wax-printing system (ModelMaker II, Solidscape, Inc., Merrimack, NH, USA)
	Carlo Reis et al., 2011 [102]	In vivo	3D-printed scaffold	PLGA/CaP bilayered biomaterial	Not mentioned
	Park et al., 2012 [68]	In vivo	3D-printed scaffold	Poly-ε caprolactone solution (PCL)	3-D rapid prototyping wax printer (ModelMaker II; Solidscape Inc., Merrimack, NH, USA)
	Obregon et al., 2015 [103]	In vivo	3D-printed scaffold	Bilayered biomaterial	Not mentioned
	Vaquette et al., 2012 [69]	In vivo	FDM + solution electrospinning	PCL	FDM, Osteopore Inc. Singapore In-house solution spinning device
	Costa et al., 2014 [91]	In vivo	3D-printed scaffold	Bilayered biomaterial	Not mentioned
	Park et al., 2014 [104]	In vivo	3D-printed scaffold	Gelatin, chitosan	Not mentioned
	Lee et al., 2014 [70]	In vivo	Layer-by-layer deposition	PCL + hydroxyapatite	Bioplotter, EnvisionTEC
	Rasperini et al., 2015 [26]	Case report	3D-printed Bioresorbable Scaffold	PCL	SLS (Formiga P100 System; EOS e-Manufacturing Solutions, Pflugerville, TX, USA))
	Sumida et al., 2015 [105]	RCT	3D-printed scaffold	Titanium	Not mentioned
	Pilipchuk et al., 2016 [106]	Preclinical study	3D-printed scaffold	PCL	Not mentioned
	Adel-Khattab et al., 2018 [107]	In vitro	3D-printed scaffold	Bioceramic	R1Series ExOne (PROMETAL, North Huntingdon, PA, USA)
	Lei et al. 2019 [108]	Case report	3D-printed bone model	Not mentioned	Not mentioned
	Bartnikowski et al., 2020 [109]	RCT	Layer-by-layer deposition	PCL	Bioplotter, EnvisionTEC, Dearborn, MI, USA
Socket preservation	Goh et al., 2015 [97]	Pilot RCT	3D-printed bioresorbable scaffold	PCL	FDM techniques (FDM 3000; Stratasys, Eden Prairie, MN, USA)
	Kijartorn et al., 2017 [98]	Prospective study	3D-printed scaffold	Hydroxyapatite granules	Projet 160, 3D systems
	Park et al., 2018 [96]	In vivo	3D-printed bioresorbable scaffold	PCL	3D bioprinting system (laboratory -made system in Korea Institute of Machinery and Materials, Daejeon, Korea)
Vertical bone augmentation	Tamimi et al., 2009 [110]	Case report	3D-printed monolithic monetite blocks	Synthetic calcium phosphates	3D-powder Printing system (Z-Corporation, Burlington, MA, USA)
	Torres et al., 2011 [100]	In vivo	3D-printed monolithic monetite blocks	A/b-tricalcium phosphate	3D-powder Printing system (Z-Corporation, Burlington, MA, USA)
Sinus augmentation	Mangano et al., 2015 [103]	In vivo	3D synthetic bone substitute	Ceramic	Not mentioned
Guided implant placement	Di Giacomo et al., 2005 [111]	NRCT	SLA surgical guides	Polymer	Simplant CSI Materialise, Ann Arbor, MI, USA
	Cassetta et al., 2013 [112]	Retrospective	3D-printed surgical guide	Acrylic	SLA surgical guide (External Hex Safe1, Materialise Dental, Leuven, Belgium)
	Pozzi et al., 2014 [113]	Clinical trial	SLA surgical guides	Acrylic resin	Nobel Procera, Nobel Biocare, Zurich, Switzerland
	Stübinger et al., 2014 [114]	Prospective	3D-printed surgical guide	Polymer	Astra Tech AB, Mölndal, Sweden
	Shen et al., 2015 [115]	RCT	SLA templates	Acrylic	Geomagic, version 10.0, Geomagic, Research triangle Park, NC, USA
	Verhamme et al., 2015 [116]	Prospective	3D-printed surgical guide	Not mentioned	NobelGuide (Nobel Biocare, Gothenburg, Sweden
	Xu et al., 2016 [117]	In vitro	SLA surgical guides	Acrylic	Conne×350; Objet, Rehovot, Israel
	Bernard et al., 2019 [118]	RCT	SLA surgical guides	Acrylic	Simplant; Materialise Dental, Waltham, MA, USA

## Data Availability

Not applicable.
